# On climate order: a policy brief

**DOI:** 10.12688/f1000research.124603.1

**Published:** 2022-09-28

**Authors:** Rui Feng

**Affiliations:** 1 College of Environmental and Resource Sciences, Zhejiang University, Hangzhou, 310058, China; 2 State Key Laboratory of Clean Energy Utilization, Zhejiang University, Hangzhou, 310027, China

**Keywords:** Climate change; Hobbesian analysis; Westphalia; Geopolitical imperatives, Balance-of-power, climate policy

## Abstract

**Background:** Climate change, largely triggered by human-induced greenhouse gases (GHGs) emissions, seems unstoppable. There was a strong rebound of anthropogenic emissions of CO _2_, the preponderant GHG in terms of contribution to global warming, around the world after the COVID-19 lockdown. Also, there is still no widely accepted international treaty on curbing the anthropogenic emissions of CH _4_ and N _2_O, the second and third predominant GHG, respectively, so far. Thereby, *prima facie*, in respect to mitigating climate change, currently, humans have no aces up their sleeves. It seems that current temperature rise is not high enough to take alarm until the occurrence of tipping point.

**Policy:** Climate-related international treaties, such as 2016 Paris agreement, are compromises among conflicting geopolitical pressures. However, currently, the climate treaties show little mandatory binding force on the signatories who are able to violate and then get off scot-free, thus may end up like a nostrum. Throughout the European history, I find that the only way, if at all, to achieve the peace or obedience of a treaty is *via* balancing powers, embodied in Bismarck’s *Realpolitik* of Germany and Richelieu’s *Raison d'état* of France. Similarly, the Chinese history in East Asia proved the significance of unadulterated ideological neutrality and Darwinian adaptability in the kaleidoscope of evolving circumstances in maintaining order and enforcement of international treaties through balancing the power of rivalries to constrain ever-recurring challengers for equilibrium.

**Recommendations:** A successful policy needs to make a thorough analysis of all relevant factors to form a long-term strategic notion. Then, statesmen need to distill an array of nebulous, always contradictory options into a tenacious, controllable direction. Thereby, I suggest that, for better curbing global warming, climate agreements or climate club be incorporated into an overall geopolitical framework among the international communities.

## Introduction

The vicissitude of humanity has waxed and waned through the coaster ride of history. Every age has its leitmotif and every generation has their own focal points and challenges. In the young twenty-first century, along with food security, terrorism, public health and hygiene, and poverty, climate change as a shot-across-the-bow of our generation has become an inveterately arising crisis. Hence, the world had been awaiting an agreement by countries which could be in a position to hold a global vision to mitigate the rapid climate change. As an upshot, there was the 2016 Paris Agreement ( UNFCCC, 2020), aimed at keeping temperature increase below two degrees Celsius compared to the preindustrial level. After its ratification in 2016 ( Clarke and Searle, 2021), the nationally determined contributions (NDCs) and the non-mandatory long-term low greenhouse gas emission development strategies (LT-LEDS) had been submitted by signatories by 2020 to show their internal aspirations and planned measures towards mitigating climate change, striving for transforming the efforts for abating global warming from headlines to trend lines and translating conceptual recognition into concrete acts.

Even though the national strengths are in constant flux, the Paris Agreement has proved a possibility that distinct countries can cooperate on common goals through quasi formal mechanisms. Where there is an agreement, there is a foreseeable benefit; where there is a benefit, there is an order to grant and maintain it agreeable, attainable, and sustainable for the players. Thereby, the overarching questions arise: is this order established for abating climate change entire unto itself or integrated into a wider international system? And with deepening cooperation on climate change among countries, which order’s norms could prevail on the scene?

In this study, I examine the 2016 Paris agreement, the one that has not been forged in the school of hard knocks. A climate treaty needs to take the multiplicity of different interpretations on climate change among countries with distinct historical experiences into consideration and sublimate a variety of societies with diverse national interests into common search for curbing GHGs emissions. The vitality of climate treaty is reflected in the balance it strikes between changes in national power caused by climate change and that by the adoption of GHG emission reduction policies, which depends on the relative method of calculation given to each, indicating the necessity to form congruent perceptions of how the policies on GHGs reduction would affect the geopolitics in practice for a society. Climate policy is a cost-and-benefit analysis.

According to history, when confronting potential threats that may occur, most countries preferred waiting until a major threat took tangible, specific shape. In the history of Europe, *Realpolitik* promoted by Otto Eduard Leopold von Bismarck (1815–1898) and *Raison d'état* proposed by Armand-Jean du Plessis de Richelieu (1585–1642) had the historic roles as the standard-bearer of German and French statehood, respectively. Both Bismarck and Richelieu believed that there was no superior principle that can constrain the inner desire of a country for power and development, and an international agreement could only be attained by correctly evaluating the components of national strengths.

### Hobbesian analysis on climate order

Thomas Hobbes (1588–1679) firstly developed a biological-psychological description of human nature based on inherent self-affirmation and reason, from which the starting point of modern politics might lie ( Xifra, 2017). Hobbes argued that the perpetual and unchanging humanity accords the politics and order timeless necessity ( Ewin, 2001). John Locke (1632–1704) and Jean-Jacques Rousseau (1712–1778) also traced a similar viewpoint that timeless self-love frames the direction of social evolution. Sown in the nature of man, the principles and legitimacy through the evolution of human society have been established, so is the international order. On the basis of self-love and intrinsic yet frail sense of reason in essentially Hobbesian analysis, conventional order has seen peoples and nations—regarded as inherently competitive with a Sisyphean character—two intrinsic purposes: survival and development, which are labelled as the core interests of all living organisms, of which the inner dynamic of self-preservation is the fundamental driving force. Natural selection favors the genes that manipulate the world to ensure their own reproduction and altruism is not a part of our biological nature ( Dawkins, 1989). The strength of a people or a society is the principle of order with which it most efficiently marshals its limited resources. When two nations meet, a testing of limits and strengths is inevitable and then a broader range of order may emerge as a corollary of collisions between two unparallel egos. Goodwill and neutrality are required to reach an agreement or a consensus peacefully. But in international politics, the imperatives of geopolitics override the pursuit of philosophical loftiness or moral purity ( Kissinger, 2015), rendering a pragmatic dimension on actions. So as to the issue of global warming, leadership does not need to search for an idealism at the expense of lowering a country’s aspiration for survival and development. The ultimate motivation to curb climate change is distilled from an ecumenical calculation of national interests, not ethical restraints. In this view, the possible gain and loss from climate change are integrated into a panoramic analysis of national interests. What matters is how to incorporate the climate change with the other factors that impact on the survival and development of a nation into a design to make an overall guideline for policy-making. After all, the only sustainable foundation of policy of great powers is egotism and there is no reciprocity for sentimental policies. No country would do anything regarded as the rewards for others’ sacrifice. *Entente cordiale* is strictly due to utility, not gratitude.

## The existing orders in history: implications for climate order

### Wilsonianism and the League of Nations

The Wilsonianism proposed by Woodrow Wilson was aimed to seek for world peace and universal harmony by cultivating shared principles and standards to bridge the gaps between realism and idealism. However, to some extent unmoored from Hobbesian analysis and taking refuge in wishful thinking, Wilsonianism, which mainly focused on international law, humanitarian objectives, and goodwill, ran into the sand due to its disconnection from a sense of geopolitics ( McDougall, 2015). In other words, mortgaging futures on the cultivated goodwill is quixotical and hardly successful, because the core interests of a nation cannot be respected if itself is unable or unwilling to fight for them. The past is the best mentor for the future. The League of Nations (1920-1946) failed to promote the disarmament in 1920s and 1930s ( Eloranta *et al.,* 2011), because it failed to offer security guarantees for memberships due to a lack of resolutions for enforcements.

The downfall of Wilsonianism and the League of Nations was because policies followed public opinions which viewed agreements can be maintained by disinterested consensus and harmony is the natural condition of humans, thus often departing from Hobbesianism. For another case, Klemens von Metternich (1773-1859) could not stop the unification of Germany by Prussia, the most powerful geopolitical opponent of Austria, because his policy concentrated too much on principles but failed to maintain an equilibrium of power in central Europe. In a conventional Hobbesian analysis, when expecting the worst from human and making a virtue of necessity, decision-makers rarely find themselves disappointed. Since climate change policy has no strong geopolitical binding force, the outcome may end up like that of the Wilsonianism-based League of Nations.

**Figure 1.**
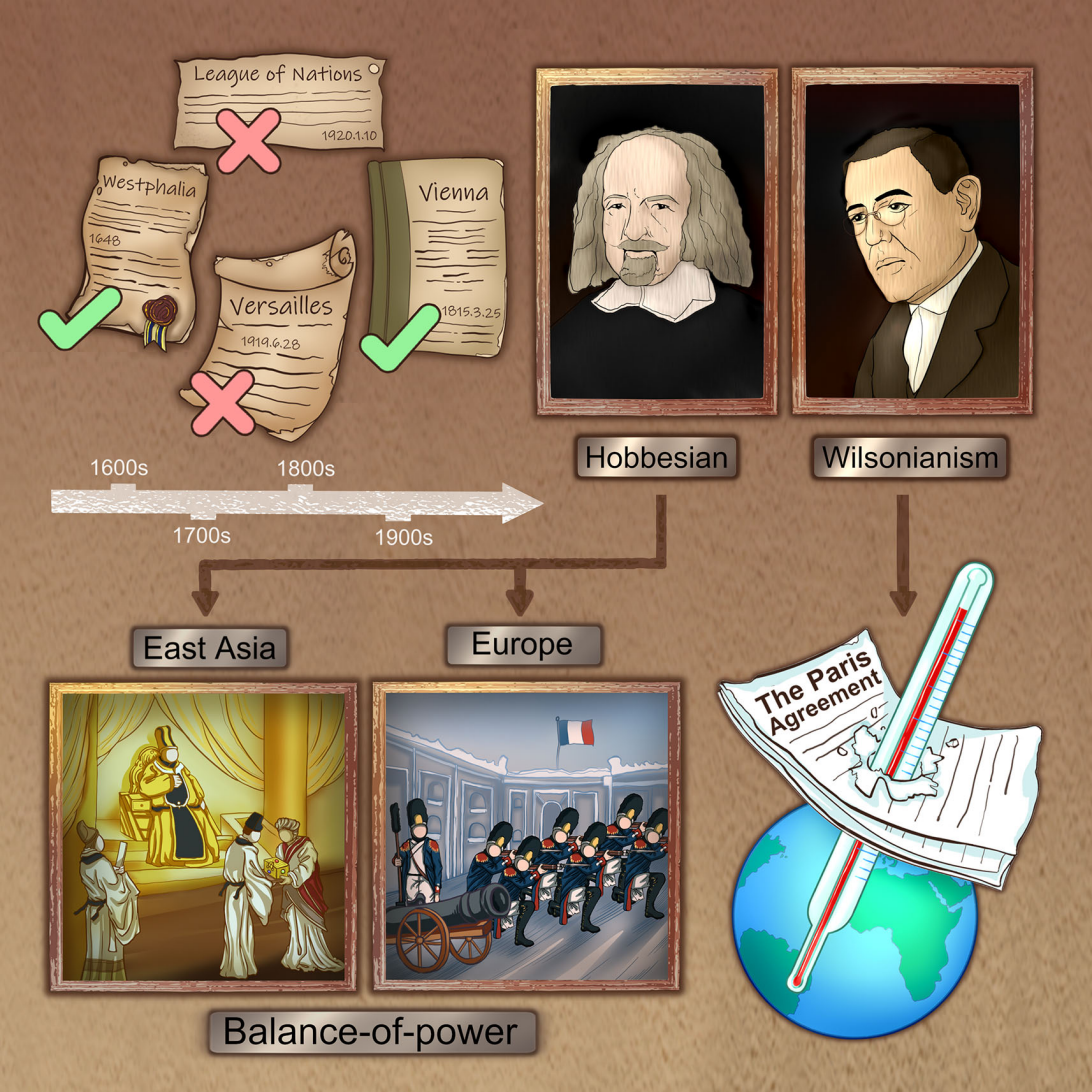
International orders that had prevailed in history: Following the doctrines of balancing the power, the 1648 Treaty of Westphalia and 1815 Treaty of Vienna ended in obedience and peace; on the contrary, the League of Nations and 1919 Treaty of Versailles failed to balance the power and ended in disobedience and war. The core ideas of the balance-of-power in Europe and the Confucian hierarchy in East Asia are basically similar to the Hobbesianism while the climate agreements are more likely derived from Wilsonianism.

Throughout the history of humanity, two types of international order have been established and prevailing regionally, shown in Figure 1. Following the doctrines of Hobbesianism and reaching a strategic equilibrium, the 1648 Treaty of Westphalia and 1815 Treaty of Vienna succeeded to maintain peace. On the contrary, following the doctrines of Wilsonianism and failing to balance the power, the League of Nations and 1919 Treaty of Versailles ended in war.

The lodestar of an order that is supposed to be sustainable contains three elements: it defines the minimum condition of the survival of a nation, its scope must contain the minimum *arrière pensée* of a nation for its own development, and it must assess the maximum aspiration of a nation for pursuing its national interests. Meanwhile, a treaty should reflect a sense of shared purpose, a recognition of common threat, and the ability to gather strengths for joint actions.

### Westphalian balance-of-power in Europe

The treaties of Westphalia in 1648, which was aimed to reduce the risks of exhaustion by excessive consumption of human resources and drain of strength in vain by congenitally competitive nature of man in the incessant competitions, had marked a turning point in the history of Europe ( Kissinger, 1994), verifying that peace and sovereign equality can be established upon balancing rivalries and appealing to all participants into a shared search for establishing order. In Westphalian principle, a system can be constrained in a narrow range of equilibrium through limiting the more powerful competitors by spontaneous alliance of counterweighing forces. Since the powers are in constant flux, the ingenuity of Westphalia-style order embodies in its pathway: it is essentially procedural, not substantive ( Kissinger, 2015). In this view, adroitly maintaining the equilibrium and deftly shifting in coalescence as a balancer towards the weak side among powers is the central strategic purport of foreign policy for a nation, effectively preventing the emergence of a Machiavellian approach by the strongest nation. The kernel of this kind of order is epitomized by its fluidity and pragmatism. Even though some may point out that equilibrium is the temporary consequence of an intermeshing of self-serving intentions, history has revealed that the eras of the successful enforcement of balance-of-power were the most peaceful time in Europe. For example, the Congress of Vienna in 1815 ushered in a century of comparative stability in Europe, because it achieved the geopolitical equilibrium in Central Europe. The success of peace-keeping in the 1648 Treaty of Westphalia indicates that only by achieving a geopolitical balance-of-power can a treaty be implemented and obeyed. Since the damages of climate change is too difficult to make similar evaluation among countries, the willingness to vindicate the GHGs emissions too multifarious, and imposing punishment for violation is highly likely out of the question, the climate agreements only spin a web of moral restrictions without geopolitical restraints. Thus, the weakness of climate treaty is the interests of countries in a changing climate are not uniform and the enforcement is rarely seamless, leading to inaction whose alibi may be circumspection. The signatories of climate treaties may highly possibly witness the withdraw of the most powerful member who feels its own survival will not be much impacted in a warming climate. Actually, on November 4, 2019, the United States announced its decision to withdraw from the 2016 Paris Agreement.

### Confucian hierarchy in East Asia

In East Asia, as nations shared the devastating experience of the conflicts derived from misunderstandings, they were committed to preventing the recurrence of warfare. Since Asian countries had more distinguished languages and cultures than Europe, their predominant goal was to find a way to clearly understand the real intention of the distinct neighborhoods to prevent high-wire acts. Overcoming the obstacles in the communications of the continent's diverse peoples, ancient China had established a tributary system to foster an order based on the hierarchical concepts of Confucianism. The essence of this tributary system is not so much material as ritual and conceptual. In fact, China often gave more gifts to the tributary countries than tributary countries offered to China ( Kissinger, 2012). China’s goal was neither to acquire economic gains nor to seek domination, but to reduce the possibilities of potential conflicts triggered by misconceptions or incomprehension of the surrounding nations that had disparate cultures and to promote deference from its recalcitrant neighbors. The prerequisite for countries that chose to join this tributary order was the acceptance of the peaceful resolution of disputes with a shared etiquette of fair conducts among all participants, acting as a decisive catalyst to satisfy all sides' amour propre. China regarded itself as the patriarch of this familial hierarchy with a role in keeping the etiquette functioning smoothly. The genius of this kind of order whose courtesy emphasizes reciprocity is that it’s procedural, not substantive, softly sending all players to find its appropriate place in the system, ensuring dissatisfaction of countries in the East Asia lower than the level at which some may seek to overthrow the established order, and preventing the emergence of a geopolitical rival with the national strength close to China. Through this international order, China had prospered for a long time in history. But when China became weak, the equilibrium of power was broken at the periphery of China and this tributary system inevitably collapsed. In essence, this Chinese-created international system is a balance-of-power in all but name. Similar to the lessons from the 1648 Treaty of Westphalia, the international order in East Asia proved that only by realizing the balance-of-power can a treaty be sustained. The Confucian hierarchy is the Chinese version of *Realpolitik* and *Raison d'état*, for assembled therein was similar to the essence of Hobbesianism, indicating when making climate policies in East Asia, geopolitical imperatives should be considered.

## Climate order

### Innate character

The lessons from Wilsonianism, the League of Nations, the Westphalian balance-of-power, and the Confucian tributary hierarchy indicate that agreements or consensus can be only made possible upon the pursuit and fulfillment of self-interests of each participant and that a sustainable order can only be founded in a more procedural and less substantive framework that guarantees, if not boosts, the internal core interests of major players.

In this view, the precondition of the success of the 2016 Paris Agreement depends on a thoroughly, accurate analysis on each nation’s threshold for threats of global warming. Order formation is where the past meets the future. The northern nations, with a cold climate, may prefer to withhold explicit commitments and wait for a major domestic threat from climate change to take specific shape. For example, Russia did not join the Paris Agreement until September 2019. For those nations that have a narrow margin of safety in a warming age, they may find their survival and developments in danger from a smaller degree of climate change than that which causes the other countries to be alarmed. The lifeblood of creating climate policy is reflected in the balance it strikes between the prediction of what the impending risks of climate change are and the evaluation on how much it costs to mitigate it. Each nation’s own methods of calculation given to each variable are the fulcrum to formulate policy. If the scale is tilted towards mitigation, actions may obtain an extent of spontaneity. However, the perspectives on national interests (survival and development) are too diverse and historical experiences too distinct among cultures. For instance, where American envisions infinite optimism, Russian may experience stoic forbearance.

The maximum temperature increase for which different countries can tolerate diverge. Variant degrees of tolerance for climate change among nations may lead to the countries with high threshold to use its endurance as a bargaining chip to render a broad berth for practical necessities towards their own geopolitical advantage. Brinkmanship may become an end result of instituting policies. Thus, climate-related policies are inclined to be integrated into an overall assessment of geopolitical realities, under the framework of either Europe’s Westphalian equilibrium or East Asia’s Sino-centric hierarchy. Since the geopolitical power is in constant flux, the climate order is not so much as a Newtonian concept of interlocking mechanical clockwork regularity as a Darwinian evolution of the survival of the fittest.

### The principle watcher in climate order

Global agreements on climate issues can be traced back to the 1997 Kyoto Protocol, but up to now, no considerable coordinated mitigation has come about yet ( Nordhaus, 2021). The United Nations (UN) plays a role as a platform whose framework provides a procedural, not substantive pathway to blunt the edges of controversies among nations. Thereby, a new order that has combined Europe’s Westphalian equilibrium and East Asia’s Sino-centric hierarchy has been established. Five predominant powers, inclusive of China, the United States of America (USA), Russia, the United Kingdom (UK), and France, have been selected as the permanent members of the United Nations Security Council, balancing each other. When these five great powers pursue their own interests with restrain, their intermeshing ambitions can reach an equilibrium, and then possibly an agreement can be fulfilled. Inside the top UN hierarchy, the five permanent numbers are in a Westphalia-style balance-of-power. Outside the top of hierarchy, they act as the patriarchs under the banner of the Charter of the United Nations, mastering the art of adopting a calibrated combination of rewards and punishments to settle disputes and abate conflicts.

A new concept of the climate club has been proposed recently to combat free-riding countries, as the participants in the club supposedly are obliged to take a uniform tariff on all imports from non-club nations ( Nordhaus, 2021). While highly possibly analogous to the test for Wilsonianism-based League of Nations, the trial on the climate club may never be whether the club members are able to enshrine or consecrate the treaty on curbing climate change *via* amply elaborate rules with a wide range of signatories. The fundamental test is what to do if these rules are violated, challenged, or even manipulated against the core ideas of the club and how to execute it. The fabric of the entire planning edifice may unravel and be out of kilter even if just one strand were drawn out. Hence, in a systematic and conceptual manner, a precondition for the success of the climate club is that those who have been required to take the lead in checking the disobedience of its principles view the dangers of climate change in the same light and have the willpower and capability to mete out the punishments to anyone who violates the principles.

Thus, in this climate club, the guardians of the principles of climate order are of great importance, especially as the power and willingness of countries to maintain these principles are in an ever-changing, fluid equilibrium. Not only proscription is enough, but also prevention is needed by forces. An agreement is workable only if it reflects a balance-of-power. The failure of the enforcements of collective security established from the concepts of Wilsonianism and the League of Nations was because the definition of violation was so vague and the reluctance to take joint action so strong.

Thus, not to put too fine a point on it, doubts arise on what action to take if a member of the Paris Agreement is so determined to disobey the principles, and if the democratic public consider that punitive measures by forces are against current international laws in the framework of UN. Can the rules and principles of the climate club themselves build an order, or are they adding a scaffold unto the geopolitical structure that currently exists? Will the climate order construct a live-and-let-live system? In case of violation, confrontation, or didactic cooperation, which may prevail? If an international framework for cooperation is only made possible when it’s more procedural and less substantive, is the criterion for selecting memberships on the basis of their power or a low threshold of climate change impact? What role will the climate club play when geopolitical imperatives transcend it? How will it compensate for the sacrifices made by countries with high thresholds of climate change risk under the shadow of the future?

As an old saying goes that “history does not repeat; it rhymes” ( Kissinger, 2012). In a Hobbesian analysis, a contest over the preeminence in an order is always predictable and foreseeable. Matters of specific tariff concessions or negotiating norms are not as important as contesting for the leadership over the nature of the climate order. As discussed above that the principles of climate club will be highly likely integrated into a global geopolitical structure, not as a separate order, carbon neutrality is a matter of price, not principle, for the essence of climate orders is based on the geopolitical interests. And a climate institution should consist of a core group of hard-nosed countries to obtain a certain degree of hegemonic stability. Only by this way, a country would have a reason and willingness to accept an international system in which to anchor itself.

## Ambiguity in the notion and scientific basis of carbon neutrality

The 2016 Paris Agreement is the first widely accepted international treaty to introduce the notion of carbon neutrality. But the concept and definition of carbon neutrality are nebulous. Whether the term ‘carbon neutrality’ only includes net zero emissions of CO _2_ or contains other types of GHGs is unclear. Common GHGs include CO _2_, CH _4_, N _2_O, NF _3_, SF _6_, hydrofluorocarbons (HFCs), and perfluorocarbons (PFCs). According to Le Ravalec *et al.* (2022), carbon neutrality means that anthropogenic GHGs emissions are offset by reduced emissions and increased sequestrations, thus striking a balance between discharges and sinks. European Union (EU) has planned to realize climate-neutral by 2050, indicating that the EU takes the total GHGs emissions into account ( European Council, 2021). However, currently, China only focuses on the net zero emissions of CO _2_ and does not take the emissions of other types of GHGs into considerations ( MEE, 2021).

Another discombobulating unclearness is the definition of net zero emissions. The question arises of how to evaluate whether carbon neutrality is achieved. According to Huovila *et al.* (2022), carbon neutrality means that CO _2_ emissions are balanced by local sinks. The categories of CO _2_ emissions and sinks can be divided into natural and anthropogenic sources. Recently, a report from the Intergovernmental Panel on Climate Change (IPCC) showed carbon neutrality means human-induced CO _2_ emissions are counterbalanced by anthropogenic CO _2_ removals over a specified time horizon ( IPCC, 2021). However, so far, there is still no official confirmation that whether carbon neutrality includes natural emitting sources and natural sinks or not. Anthropogenic CO _2_ sinks are inclusive of two pathways: industrial and ecological. Industrial removals refer to carbon capture, utilization, and storage (CCUS) technologies ( Chen *et al.,* 2022). Ecological removals refer to the absorption of atmospheric CO _2_ through protection, restoration, and sustainable management of ecosystems ( Lu *et al.,* 2022; Griscom *et al.,* 2017), such as afforestation and silviculture. However, because these artificially cultivated trees have been afforested all over the country, often mixed with natural forests, it’s hard to assess their total carbon sequestration abilities.

The lodestar of strategy-making process in statecraft involves five successive, ironclad elements in sequence: firstly, making a rational, pragmatic calculation of long-term trends in the grand schemes of things on the basis of national interest in order to handle the rough patches of confronted challenges; secondly, meticulously drawing a strategic landscape and then detachedly exploring the range of choices and means of tactics; thirdly, sweepingly calibrating the internal abilities of a country and then cautiously questing for any underlying congruence and confluence of interests around the world to establish potential international cooperation under the banner of mutual respect and symbiosis; fourthly, prudently opting the most eminent practical pathway of implementation, swinging into a tentative action via daring execution to outline the maximum real-life capacity, and then warily assessing the division, if at all, between the anticipatable and the achievable; fifthly, progressively, scrupulously adjusting to a more attainable, crystalized stance through nuance and osmosis to deftly realize the optimal objectives at the minimum costs amid the kaleidoscope of changing circumstances ( Kissinger, 1994, 2012).

The climate issue involves two aspects that are unusual compared with the other crises. Firstly, it affects almost every country and every species in the world, thus GHGs emissions show a pattern of global externality; secondly, it represents a problem that a single country alone cannot solve ( NASEM, 2017). Hence, mitigating global warming needs international cooperation. However, even though the long-term trend is clear and the strategic landscape is certain as our planet keeps heating up, but since the definition of carbon neutrality is vague, the policy-making process could not be fully completed. Ambiguity at times is the lifeblood and fulcrum of policy-planning in order to entitle the battle-hardened decision-makers enough elbowroom to create margin of flexibility, blunt the edges of tangential factional disputes, gain freedom of maneuver and degree of resilience, and balance powers ( Kissinger, 1994, 2015). However, upon tactic level, a commonly-accepted definition would furnish the impetus for decisive, unified, and audacious actions, and the *de facto* effective policy formulation would not be come forth if the scientific notion, the bedrocks of policy-making, began to diverge.

## Implications for formulating climate policies

Climate policies are refracted and filtered through the prism of national interests. Deviated from geopolitical realities, the feasibility of green growth strategies for the economy has been frequently doubted ( Franssen and de Wilde, 2021), reflecting the current dilemma of many countries around the world: how to balance their carbon emissions reduction and economic development, and how to seek to navigate the narrowest of passages between economic development and greenhouse gases (GHGs) reduction. Reportedly, the realizations of the commitments of emission reductions by countries made in the 2016 Paris Agreement can keep the global warming below 2 °C ( Meinshausen *et al.,* 2022; Hausfather and Moore, 2022). However, as a *fait accompli*, with the strictest practicality and most level-headed realism, yet falling far short of their rhetoric, the 20 largest economies around the world have not fulfilled the letter of their pledges for the reduction of GHGs emissions and instead have focused more on economic developments, especially during the COVID-19 period ( Nahm *et al.,* 2022), indicating the facts that their demands for economic development have transcended their internal desire for climate change mitigation ( Liu *et al.,* 2022). The reality of the 2016 Paris Agreement seemed emphasizing atmosphere over substance, showing that most countries have deemed that sacrificing economic developments to lower climate change is pyrrhic. A predominant reason for this judgement is a sense of doubts on the unfounded and undemonstrated impact of global warming on human welfare ( Lomborg, 2020). Also, some climate models predict that the future will get too hot too fast ( Hausfather *et al.,* 2022), undermining the credibility of climate science ( Voosen, 2022).

In the resulting maelstrom, evidently, global CO _2_ emissions in 2021 were almost equal to that in 2019 ( Davis *et al.,* 2022), exhibiting a strong rebound of anthropogenic emissions from the COVID-19 pandemic and experiencing the largest-ever yearly increment in the absolute term of anthropogenic CO _2_ emissions compared to that of the previous year (IEA, 2021). The understanding that insufficient actions will never lead to political debacle results in political flexibility on climate targets ( Geden and Löschel, 2017). *De facto*, climate order is an outgrowth of a balancing of risks and rewards in terms of geopolitical considerations and calculations of the national interests. Again, reality proves that international agreement is built on quicksand if it deviates from the doctrines of balance-of-power on the basis of Hobbesianism which embody in the German *Realpolitik* during the times of Bismarck and in the French *Raison d'état* during the times of Richelieu. Actually, departing from Bismarck’s *Realpolitik* and Richelieu’s *Raison d'état*, and ignoring current geopolitical imperatives, the milk-and-water climate agreements present an ominous vista and a myopic form of risk-taking.

With bite on granite, according to a report from World Meteorological Organization ( WMO, 2022), there is a 50:50 change that the global annual average temperature for at least one of the next five years will reach 1.5°higher than the average over the years between 1850 and 1900. The visionary statesman Henry Kissinger once stated “No one eats with impunity from the tree of immortality” ( Kissinger, 1994). The ends justify the means, not the other way around. The lessons of history have told us that the geopolitical imperatives are the main driving force to reshape the road towards net zero ( Thompson, 2022). Carbon neutrality is hard to realize when the geopolitical realities have been ignored. Evidently, the willingness of voluntary corporate actions for mitigating global warming by companies has been frequently questioned ( Bjørn *et al.,* 2022a,b). Several world-famous companies or institutions, inclusive of Shell, British Petroleum (BP), Equinor, and International Energy Agency (IEA), introduced incompatible energy development pathways with that recommended for Paris agreements 1.5 °C goal ( Brecha *et al.,* 2022). Ratification of a climate treaty alone but neglect of the geopolitical realities never can be a sort of *deus ex machina* to slow down climate change. After all, for now, before the occurrence of the tipping point which is more obvious to identify in *ex post facto* retrospect than when we are moving towards it, carbon neutrality, for most countries, is more a price of bargaining chip for playing out geopolitical gambit, less a principle. Ultimately, in the international community, possession of power is nine-tenths of the law. To the victor belong the spoils, which is the rights to interpret the definition of carbon neutrality. Without meeting the geopolitical imperatives or considering Machiavellian realism, complying climate treaty is not a natural condition of man, but a temporary oasis in a perilous world where obedience could only be sustained by permanent vigilance.

Top climate scientists do doubt that countries around the world will effectively control global warming ( Tollefson, 2021). Decades from now, historians will possibly hold a fact-finding debate on who must bear the responsibility for completing the construction of the doomsday machine that has led us to irreversible global warming and for blowing away the house of cards assembled by the most far-sighted scientists who have an unerring eye for the long-term future trend.

Yet I guess at the time of the start of this future debate, no one country will be singled out to blame for this mad dash towards climate change. Each of them contributed its own quota and did so with the carefreeness which would never again be possible when the negative consequences of global warming they have triggered enter the shared memory of humanity. In preparing themselves for the worst-case scenario, they facilitate to make it a reality and are hoisted by their own petard. However, taking the future with a grain of salt, I deem that when temperature keeps going up with the passage of time to the degree that will impact the geopolitical imperative of most countries, the gains for lowering global warming transcend that for pursuing social and economic developments and major economics will place a greater premium on curbing GHGs emissions without falling by the wayside. Reportedly, the time to take alarm for human survival as the tipping point is when the increase of global average temperature exceeds at most 5 °C above preindustrial levels ( Lenton *et al.,* 2019; O’Grady, 2022; McKay *et al.,* 2022).

## Actionable recommendations

My suggestion is that when policymakers are formulating climate policies, they must consider the geopolitical realities. Evidently, without taking geopolitical imperatives into considerations, the endeavors to mitigate global warming are limited in the effectiveness ( Thompson, 2022). For instance, China used its rare earth mineral resources as a weapon to weaken its geopolitical opponents. Rare earth minerals are essential for the development of renewable energy. Meanwhile, due to the Taiwan crisis in August 2022, driven by a realistic evaluation on national interests and internal capabilities, China announced the suspension of Sino-US climate talks. Therefore, if only history were more forgiving, it seems that the world is still waiting for the occurrence of the tipping points.

When an international order cannot be actualized by consensus or enforced by power, chaos may occur in a dehumanizing way. Since the tolerance of temperature rise for different countries differs, confronting may fill the vacuum between the different tipping points of two countries. From the perspectives of Machiavelli and Clausewitz, in the battle for survival, a nation may use the GHGs emissions as a weapon to weaken its geopolitical opponents who are more sensitive to climate change.

Once Bismarck wrote in his poet: “That which is imposing here on earth has always something of the quality of the fallen angel who is beautiful but without peace, great in his conceptions and exertions but without success, proud and lonely.”

## Data availability

No data are associated with this article.
