# Supplementary material for: Climate politics in global Hobbesian Jungle
Source: F1000Res. 2024 Jun 13;11:1109. Originally published 2022 Sep 28. [Version 2] doi: 10.12688/f1000research.124603.2 (PMC11294801; doi:10.12688/f1000research.124603.2)
Supplement: Peer review history

# Reviewer report: Reviewer response for version 2

**DOI:** 10.5256/f1000research.167400.r290894

**Published:** 2024-08-01

**Authors:** Md. Emran Hossain

**Affiliations:** 1 Texas State University, San Marcos, Texas, USA; 2 Bangladesh Agricultural University, Mymensingh, Dhaka Division, Bangladesh

No further comments

Does the paper provide a comprehensive overview of the policy and the context of its implementation in a way which is accessible to a general reader?

Yes

Is the discussion on the implications clearly and accurately presented and does it cite the current literature?

Partly

Are the recommendations made clear, balanced, and justified on the basis of the presented arguments?

Partly

Reviewer Expertise:

Environmental and Energy Economics

I confirm that I have read this submission and believe that I have an appropriate level of expertise to confirm that it is of an acceptable scientific standard.

# Reviewer report: Reviewer response for version 1

**DOI:** 10.5256/f1000research.136811.r219155

**Published:** 2023-11-13

**Authors:** Elzbieta Wojcik-Gront

**Affiliations:** 1 Warsaw University of Life Sciences, Warsaw, Poland

Climate change is a significant problem, and there are no strong rules in place to address it effectively. Some treaties and agreements lack the necessary mechanisms to ensure compliance by countries. To address this, the author suggests that climate agreements should be integrated into a broader international framework involving cooperation among multiple nations.

 The author argues that there is no single overarching principle that can effectively limit a country's pursuit of power and development. Achieving international cooperation depends on a precise assessment of each nation's power. Climate policy is influenced by various national interests, historical experiences, and geopolitical realities, often striking a balance between risk assessment and mitigation costs. The current focus on carbon neutrality primarily occurs at the national level, even though climate change is a global issue that necessitates international collaboration. International cooperation is vital for mitigating global warming. Policymakers crafting climate policies must consider geopolitical factors to enhance their effectiveness.

 This analysis offers an intriguing perspective on the challenges of reaching a consensus on climate change policies. Undoubtedly, it provides valuable insights that can resonate with a wide readership interested in the topic. Furthermore, it has the potential to influence policymakers' viewpoints on the matter.

Does the paper provide a comprehensive overview of the policy and the context of its implementation in a way which is accessible to a general reader?

Yes

Is the discussion on the implications clearly and accurately presented and does it cite the current literature?

Yes

Are the recommendations made clear, balanced, and justified on the basis of the presented arguments?

Yes

Reviewer Expertise:

GHG emission mitigation, statistical analysis, crop modelling

I confirm that I have read this submission and believe that I have an appropriate level of expertise to confirm that it is of an acceptable scientific standard.

# Response

**Published:** 2024-06-01

**Authors:** Rui Feng

**Affiliations:** Zhejiang University, China

Thank you for your positive words toward my work. It really encourages me.

# Reviewer report: Reviewer response for version 1

**DOI:** 10.5256/f1000research.136811.r192727

**Published:** 2023-08-29

**Authors:** Md. Emran Hossain

**Affiliations:** 1 Texas State University, San Marcos, Texas, USA; 2 Bangladesh Agricultural University, Mymensingh, Dhaka Division, Bangladesh

Thank you for providing me the opportunity to review the article “ **On climate order: a policy brief** **”. **The authors have made good contributions to this paper. I have completed my assessments and the paper can be improved before recommended for indexing as follows:

1. The Abstract needs to be rather precise and conclude based on empirical evidence.
2. At the beginning of the Abstract, authors should state the research gap and contribution to the literature. The background is not justifiable currently. 
3. The introduction section needs further improvement in terms of the research gap and contribution of the study with proper background.
4. Please add the motivation and write why this policy brief is important and would be beneficial for implications.
5. The discussion on SDG and environmental sustainability linkage with respect to climate policy is missing. Authors should add in the first paragraph of the introduction. The research contribution should be enlarged.
6. Please double-check the abbreviation used in the study. I have found diverse abbreviations of a word in several places. Please make sure that authors use similar abbreviations of a word throughout the manuscript.
7. Paragraphs can be adjusted. Currently, the manuscript is in rough shape.
8. The policy implications are too general in its current form. The policies need to be aligned with the recent Government Policies and mention their scope for implementation to the countries having similar socio-economic orientations. 
9. Please add the limitations and future research directions.

Does the paper provide a comprehensive overview of the policy and the context of its implementation in a way which is accessible to a general reader?

Yes

Is the discussion on the implications clearly and accurately presented and does it cite the current literature?

Partly

Are the recommendations made clear, balanced, and justified on the basis of the presented arguments?

Partly

Reviewer Expertise:

Environmental and Energy Economics

I confirm that I have read this submission and believe that I have an appropriate level of expertise to confirm that it is of an acceptable scientific standard, however I have significant reservations, as outlined above.

# Response

**Published:** 2024-06-01

**Authors:** Rui Feng

**Affiliations:** Zhejiang University, China

Thank you for providing me the opportunity to review the article “On climate order: a policy brief”. The authors have made good contributions to this paper. I have completed my assessments and the paper can be improved before recommended for indexing as follows:

 1. The Abstract needs to be rather precise and conclude based on empirical evidence.

 Reply: Thank you for this incisive advice. I’ve rewritten Abstract to make it more precise.

 2. At the beginning of the Abstract, authors should state the research gap and contribution to the literature. The background is not justifiable currently.

 Reply: Thank you for this constructive suggestion. I’ve stated the research gap and contribution to the literature at the beginning of **Abstract**.

 3. The introduction section needs further improvement in terms of the research gap and contribution of the study with proper background.

 Reply: Following your suggestion, I have added the research gap and contribution of the study with proper background in **Introduction**.

 4. Please add the motivation and write why this policy brief is important and would be beneficial for implications.

 Reply: Following your recommendation, I’ve added Section **The essence of international politics**.

 5. The discussion on SDG and environmental sustainability linkage with respect to climate policy is missing. Authors should add in the first paragraph of the introduction. The research contribution should be enlarged.

 Reply: I’ve enlarged research contribution in the revised manuscript.

 6. Please double-check the abbreviation used in the study. I have found diverse abbreviations of a word in several places. Please make sure that authors use similar abbreviations of a word throughout the manuscript.

 Reply: I have double-checked the abbreviation in the study and make sure to use similar abbreviations of a world in the whole manuscript.

 7. Paragraphs can be adjusted. Currently, the manuscript is in rough shape.

 Reply: Paragraphs have been adjusted.

 8. The policy implications are too general in its current form. The policies need to be aligned with the recent Government Policies and mention their scope for implementation to the countries having similar socioeconomic orientations.

 Reply: I have rewritten the discussion on policies.

 9. Please add the limitations and future research directions.

 Reply: I have added limitations and future research directions in the revised manuscript.
